# Integrated mapping resolves pathogenicity of 11 *CYP21A2* variants in congenital adrenal hyperplasia

**DOI:** 10.1210/jendso/bvag127

**Published:** 2026-06-05

**Authors:** Yingtong Xu, Anna Matveeva, Flemming Steen Jørgensen, Amit V Pandey

**Affiliations:** Pediatric Endocrinology, Diabetology and Metabolism, University Children's Hospital, Inselspital, 3010 Bern, Switzerland; Translational Hormonle Research Program, Department of Biomedical Research, Faculty of Medicine, University of Bern, 3010 Bern, Switzerland; Graduate School for Cellular and Biomedical Sciences, University of Bern, 3012 Bern, Switzerland; Pediatric Endocrinology, Diabetology and Metabolism, University Children's Hospital, Inselspital, 3010 Bern, Switzerland; Translational Hormonle Research Program, Department of Biomedical Research, Faculty of Medicine, University of Bern, 3010 Bern, Switzerland; Graduate School for Cellular and Biomedical Sciences, University of Bern, 3012 Bern, Switzerland; Department of Drug Design and Pharmacology, University of Copenhagen, DK-2100 Copenhagen, Denmark; Pediatric Endocrinology, Diabetology and Metabolism, University Children's Hospital, Inselspital, 3010 Bern, Switzerland; Translational Hormonle Research Program, Department of Biomedical Research, Faculty of Medicine, University of Bern, 3010 Bern, Switzerland

**Keywords:** adrenal insufficiency, VUS, CAH, congenital adrenal hyperplasia, 21-hydroxylase deficiency, CYP21A2

## Abstract

**Context:**

Genotype–phenotype correlations in congenital adrenal hyperplasia (CAH) are often complicated by rare *CYP21A2* missense variants classified as variants of uncertain significance.

**Objective:**

To resolve the pathogenicity and structural mechanisms of 11 *CYP21A2* variants (p.L10del, p.R76K, p.E162G, p.S274Y, p.L308V, p.S373N, p.P387L, p.H393Q, p.R401G, p.R436C, and p.S494N).

**Methods:**

We integrated population allele frequency analysis (gnomAD, 1000G, and 38KJPN) with in silico structural modeling (ConSurf, FoldX, and DUET) and molecular dynamics simulations. In vitro functional assays were performed in HEK293T cells using radiolabeled progesterone and 17-hydroxyprogesterone, with results normalized to CYP21A2 protein expression.

**Results:**

The variants p.L10del and p.S494N were identified as common polymorphisms (allele frequencies >5% in specific cohorts) retaining near-wild-type activity (∼67-99%), supporting a benign classification. Variants p.R76K and p.S373N showed higher activities (∼72-92%), supporting a likely benign classification. Conversely, p.L308V, p.P387L, and p.R436C exhibited severe loss of function (<20% activity for 17-hydroxyprogesterone) and were reclassified as pathogenic (simple virilizing CAH). Structural modeling revealed that p.L308V causes steric clashes in the conserved I-helix, while p.R436C disrupts the surface hydrogen-bond network essential for redox partner docking. Variants p.E162G, p.H393Q, and p.R401G showed moderate impairment (∼40-45% activity), consistent with likely pathogenic status and nonclassic CAH.

**Conclusion:**

This study provides a potential diagnostic map for 11 *CYP21A2* variants, distinguishing severe pathogenic mutations from benign polymorphisms. Our findings refine the molecular diagnosis of CAH.

Congenital adrenal hyperplasia (CAH) encompasses a group of autosomal recessive disorders characterized by impaired adrenal steroidogenesis. Over 5 types of enzyme deficiency have been identified in CAH, of which the most prevalent form, accounting for over 90% of all cases, is 21-hydroxylase deficiency (21-OHD), which results from pathogenic variants in the *CYP21A2* gene [1]. The 21-hydroxylase enzyme (CYP21A2), a cytochrome P450 monooxygenase located in the endoplasmic reticulum of the adrenal cortex, is a key component of the steroidogenic pathway. The CYP21A2 enzyme catalyzes the conversion of 17-hydroxyprogesterone (17-OHP) to 11-deoxycortisol and progesterone to 11-deoxycorticosterone, the immediate precursors for cortisol and aldosterone [2]. A deficiency in 21-hydroxylase activity disrupts these pathways, leading to a cascade of pathophysiological consequences [3-5]. The resulting impairment in cortisol synthesis disrupts the negative feedback loop of the hypothalamic–pituitary–adrenal axis, causing a compensatory surge in corticotropin secretion. Chronic overstimulation by corticotropin leads to the characteristic hyperplasia of the adrenal glands and, critically, shunts the accumulating steroid precursors away from glucocorticoid and mineralocorticoid synthesis and toward the androgen production pathway [6, 7].

The clinical presentation of 21-OHD exists on a continuous spectrum, traditionally classified into 3 phenotypes that directly correlate with the degree of residual enzyme function. The most severe, classic forms present in the neonatal period. The salt-wasting (SW) form, resulting from a complete or near-complete loss of enzyme activity, is characterized by life-threatening adrenal crises due to cortisol and aldosterone deficiency, accompanied by severe virilization in female newborns. The simple virilizing (SV) form, associated with 1% to 5% residual enzyme activity, has sufficient aldosterone production to prevent SW crises, but the significant androgen excess still causes ambiguous genitalia in females and precocious puberty in both sexes. The nonclassic (NC) form, also referred to interchangeably in literature as late-onset CAH, is the mildest clinical presentation. It typically occurs when alleles possessing 20% to 50% of wild-type (WT) activity in vitro are paired with a severe mutation and presents later in childhood or adulthood with symptoms of hyperandrogenism, such as premature pubarche, hirsutism, and infertility [8, 9]. The severe form is associated with significant neonatal mortality, and endocrinological and metabolic evaluation as well as appropriate treatment should be initiated immediately [5, 10, 11].

The first-tier diagnostic screening is radioimmunoassay in newborn screening, testing for the serum levels of 17-OHP that is the precursor of cortisol and the substrate for 21-hydroxylase [12]. However, 17-OHP levels can be influenced by several factors, including enzyme deficiencies in the steroidogenic pathway, gestational age, birth environment, multiple courses of antenatal corticosteroids, and the sex of the infant [13, 14]. This negative predictive value can be improved by incorporating secondary biochemical and molecular screening methods. Biochemical screening includes analysis of 21-deoxycortisol in blood and urine samples using liquid chromatography–mass spectrometry, as well as measurement of steroid ratios [15]. Molecular genetic screening involves the detection of *CYP21A2* mutations in DNA extracted from the same blood used in the newborn screening [16, 17].

The genetic basis of 21-OHD is uniquely complex. The *CYP21A2* gene is located within the highly variable HLA class III region on chromosome 6p21.3, in close proximity to a nonfunctional pseudogene, *CYP21A1P*, with which it shares 98% sequence homology in exons [18]. This high degree of identity predisposes the locus to frequent intergenic recombination events, primarily gene conversions, which transfer deleterious sequences from the pseudogene to the functional gene. These events account for ∼75% of all pathogenic *CYP21A2* alleles, making molecular diagnosis a significant technical challenge [19, 20].

Current research has demonstrated a strong genotype–phenotype correlation, with *CYP21A2* mutations classified into 4 groups that help predict clinical presentation [14, 15, 21]. In mutation group 0 (null), enzymatic activity is completely abolished due to large gene conversions, frameshifts, nonsense mutations, deletions, and certain missense mutations. These are typically associated with SW classic alleles [22]. In group A, both the SW and SV phenotypes may occur, usually resulting from a low level of normally spliced mRNA produced by c.293-13A/G variant in the intron 2 region. This mutation is particularly common, found in ∼25% of mutant alleles [23]. In group B, mutations retain <5% of normal enzymatic activity and commonly linked to the SV form [24]. In group C, missense mutations reduce enzyme activity to about 50% of normal and are associated with the NC form of CAH [25].

While a strong genotype–phenotype correlation has been established for common mutations, the increasing application of next-generation sequencing in clinical practice has led to the identification of a growing number of rare variants [26]. A significant portion of these remain classified as variants of uncertain significance (VUS) in clinical databases such as ClinVar or have conflicting reports regarding their pathogenicity [27]. This diagnostic ambiguity presents a major obstacle for accurate molecular diagnosis, prediction of clinical severity, and effective genetic counseling for families affected by CAH. This study was conceived to address the critical knowledge gap for 11 such variants, p.L10del, p.R76K, p.E162G, p.L308V, p.S373N, p.R401G, p.R436C, p.S274Y, p.P387L, p.H393Q, and p.S494N (Fig. 1). These specific variants were selected by data-mining large-scale population genomic databases (such as gnomAD and ALFA) to identify *CYP21A2* missense variants and indels that have relatively high population frequencies or are frequently flagged as VUS in clinical testing yet entirely lack experimental functional validation in the literature. Our multipronged approach, integrating evolutionary conservation, structural modeling, protein stability predictions, and direct in vitro functional assays, would elucidate the molecular consequences of these variants.

**Figure 1:**
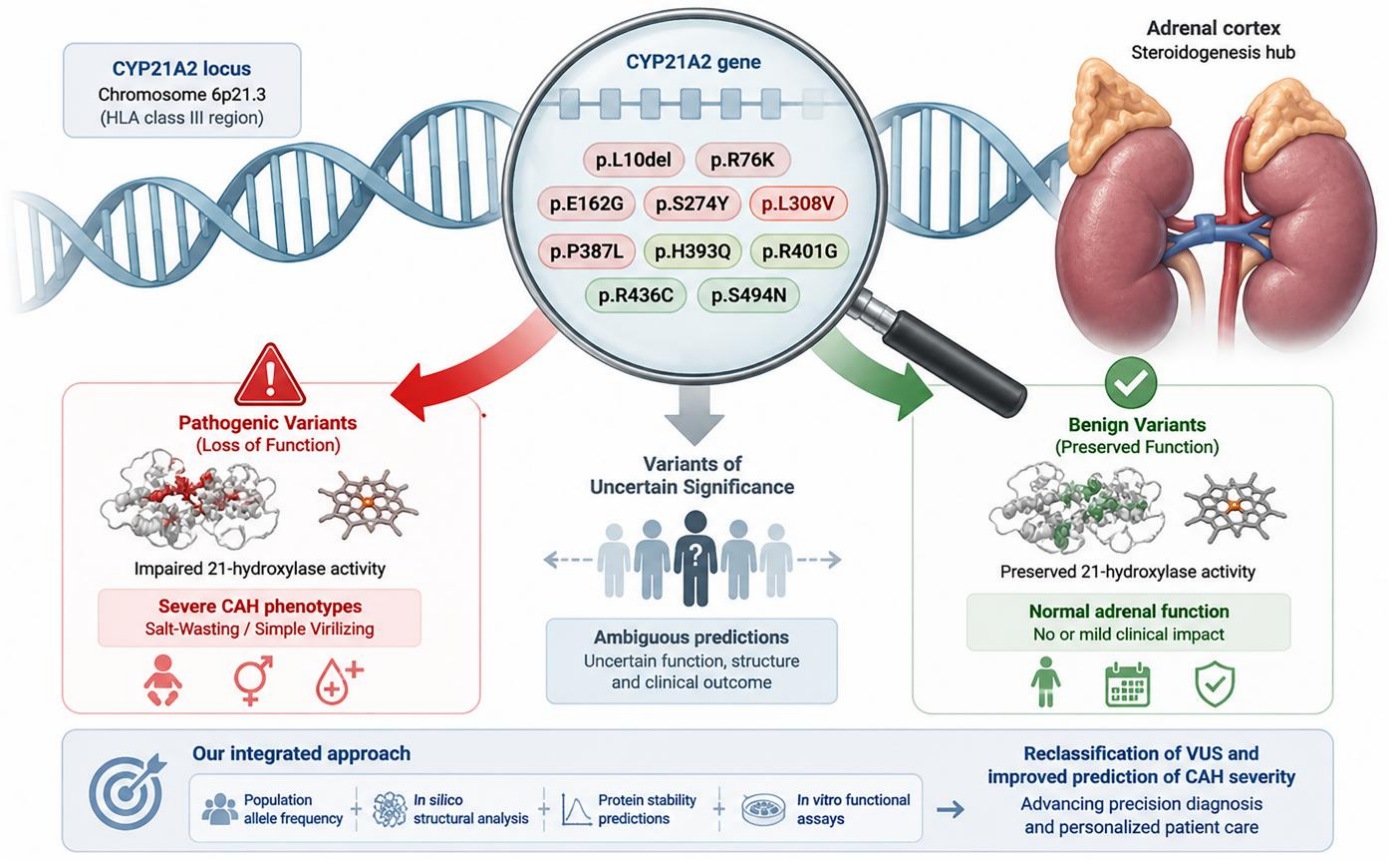
The clinical conundrum of *CYP21A2* VUS. A schematic illustration of the *CYP21A2* gene locus on chromosome 6p21.3, highlighting the eleven missense variants analyzed in this study, p.L10del, p.R76K, p.E162G, p.S274Y, p.L308V, p.S373N, p.P387L, p.H393Q, p.R401G, p.R436C, and p.S494N. We have investigated the diagnostic ambiguity facing clinicians: identifying which of these variants are benign polymorphisms (green) and which are pathogenic mutations capable of causing salt-wasting or simple virilizing CAH (red).

## Materials and methods

### Search and selection of variants in *CYP21A2*

We searched dbSNP, Ensembl, SNPnexus, and ClinVar databases for all *CYP21A2* missense mutations and indels that are characterized by high frequency in populations and were not yet studied in laboratory conditions. The frequencies for each variant were obtained from the large-scale sequencing databases, including TOPMed (Trans-Omics for Precision Medicine), Genome Aggregation Database (gnomAD) [28], ALFA (Annotation Landscape For Aligned reads) [29], ExAC (Exome Aggregation Consortium) [30], 1000 Genomes Project [31], and GO-ESP [32]. In addition, Asian population were searched through Korea4K [33] database and 38KJPN [34].

### In silico SNP analysis

The degree of amino acid conservation, chemical proprieties, pathogenicity scores, and structural damage were analyzed [35]. The conservations of polymorphic amino acids were estimated via ConSurf tool [36]. The pathogenicity scores were analyzed with the software AlphaMissense, CADD [37], DANN [38], FATHMM [39], Meta-SNP [40], MutPred2 [41], PANTHER [42], PhD-SNP [43], PolyPhen-2 [44], PredictSNP2 [45], S3Ds&GO [46], SIFT [47], SNAP [48], and SNPs&GO [49].

### Gibbs free energy calculations

To calculate the stability of *CYP21A2* SNPs, the shift of Gibbs free energy (ΔΔ*G*) was calculated using FoldX4 [50] plugin for YASARA software version 25.1.13. PDB ID: 4Y8W [51] served as a reference, and the total structure energy was minimized to repair residues exhibiting unfavorable torsion angles and van der Waals clashes. (pH 7, 298K, ionic strength 5 × 100, VdW design 2).

### Conservation analysis

To assess the significance of these variants on the evolutionary conservation, we performed an evolutionary analysis with homologous proteins by ConSurf [36]. The structure of human CYP21A2 protein deposited under PDB ID 4y8w (chain A) was used as a reference structure, and cytochrome P450 homologous sequences from *Homo sapiens*, *Bos taurus*, *Canis lupus familiaris*, *Cavia porcellus*, *Capra hircus*, *Felis catus*, *Gorilla gorilla gorilla*, *Lynx lynx*, *Mesocricetus auratus*, *Macaca fascicularis*, *Macaca mulatta*, *Mus musculus*, *Oryctolagus cuniculus*, *Ovis aries*, *Rattus norvegicus*, and *Sus scrofa* were used for conservation analysis. A multiple sequence alignment was built using CLUSTALW using the Bayesian method for the calculation of the conservation score. ConSurf scores range from conserved (magenta or 9) to variable (cyan or 1). Homologous sequences were collected from SWISS-PROT with BLAST algorithm (PSI-BLAST *E*-value 0.0001, 4 iterations) [52].

### Point mutation modeling and structural analysis

Since several residues of interest (Leu10, Ser274, and Ser494) were not present in the available crystal structures (PDB ID: 4Y8W) [51], a full-length model of CYP21A2 protein was generated using SWISS-MODEL [53] with the AlphaFold-predicted structure of CYP21A2 protein as the primary template, in combination with experimentally determined structures (5VBU.1.A, 4Y8W.1.A, 4Y8W.2.A, 3QZ1.1.A, and 3QZ1.4.A). The AlphaFold model confidence (pLDDT) indicated that Leu10 was built with high confidence (90 > pLDDT > 70), Ser274 with low confidence (70 > pLDDT > 50), and Ser494 with very low confidence (pLDDT < 50). Point mutations were introduced in Chimera 1.19 [54] using the Rotamers tool, which replaces the side chain of the selected residue with conformations derived from the Dunbrack rotamer library [55]. For each mutation, all rotamers were examined, and conformers with the highest prevalence and minimal steric clashes were selected. Local steric clashes were identified as van der Waals overlaps and evaluated visually. For cysteine substitutions, the sulfur atom (Sγ) positions were inspected, and interatomic distances between the introduced cysteine and nearby cysteine residues were measured using the distance command in Chimera 1.19 [54] Distances <5.5 Å were considered as candidates for potential disulfide bond formation. All structural visualizations and figure preparation were performed in UCSF ChimeraX 1.10 [56].

### Molecular dynamics simulations

The initial coordinates for the WT human CYP21A2 protein were based on AlphaFold model as it contained all amino acids of the CYP21A2 protein. The system was prepared by excluding the N-terminal (residues 1-29) and C-terminal (residues 486-495) regions, which are not part of the core catalytic domain. Protein structures were subjected to the Protein Preparation Procedure in Maestro [57, 58]. The final simulation system consisted of 456 CYP21A2 residues, the heme cofactor, 15 806 water molecules, and 2 Na^+^ counter-ions, for a total of 54 835 atoms. A visualization of the initial solvated system is provided in Fig. S1 [59]. Production runs of 50 ns were conducted for the WT and 9 variant proteins: p.E162G, p.H393Q, p.L308V, p.P387L, p.R76K, p.R401G, p.R436C, p.S274Y, and p.S373N with the Desmond program (v8.2.133) and comprised a default 7-step equilibration procedure followed by a 50 ns production run at 300 K using the OPLS4 force field. For each simulation 50 frames were collected and analyzed. Structures were displayed with the Pymol program (v.3.1.5.1). Structural stability was quantified by calculating the root mean square deviation (RMSD) of the Cα atoms over the course of the simulation, using the initial equilibrated structure as the reference.

### Cell transfection and CYP21A2 enzyme assays

Plasmids encoding WT human *CYP21A2* ORF sequence (NM_000500.9) and *CYP21A2* variants were purchased from GenScript (Piscataway, NJ, USA). These constructions provide transient expression in mammalian cells and are based on pcDNA3.1+/C-(K)-DYK vector with ORF sequence adjusted to a C-terminal DYK (FLAG) tag as described previously [60, 61]. A total of 6.5 × 10^5^ HEK293 cells per well were cultured in 6-well plates prior to transfection. After a 24-hour incubation period, the culture medium was replaced, and transfection was performed using 1.5 µg of plasmid DNA and Lipofectamine 2000 (Thermo Fisher Scientific, Bedford, MA, USA) according to the manufacturer's protocol. Next day the transfected cells were seeded into 12-well plates (2.5 × 10^5^ cells per well) for the steroids assay, and into 6-well plates (1 × 10^6^ cells per well) for the protein quantification. At the 48-hour mark, the 1 µM unlabeled progesterone was added to the media along with 10 000 cpm of [^14^C]-progesterone or 50 000 cpm of [^3^H]-17-OHP to serve as a radiolabeled tracer [62-65]. With the tritium-labeled-substrate cells were incubated for 90 minutes, whereas with [^14^C]-labeled ones, the incubation time was 45 minutes at 37°C. The culture medium was collected, and the steroid compounds were extracted from the media using a 1:1 (v/v) mixture of ethyl acetate and isooctane, dried, and reconstituted in methylene chloride. Separation of steroids was accomplished via thin-layer chromatography [62-64, 66, 67]. The plates were then exposed to a phosphor screen and imaged using the Typhoon FLA-7000 PhosphorImager system (GE Healthcare Bio-Sciences AB, Uppsala, Sweden). Quantitative analysis of the signal intensity was conducted using ImageQuant TL version 8.0 software (Cytiva, Marlborough, MA, USA).

The steroid conversions were used to quantify CYP21A2 enzyme function. CYP21A2 hydroxylase [^14^C]-progesterone with the formation of 11-deoxycorticosterone and [^3^H]-17-OHP to 11-deoxycortisol with the incorporation of radiolabeled atoms into these products. The percentage of the total radioactivity detected in each sample. These conversion percentages were then compared between the WT and variant forms of CYP21A2 enzyme. Data were derived from 2 replicates per condition, in 3 independent biological replicates. To account for potential differences in CYP21A2 protein levels across samples, enzyme activity was normalized based on relative protein expression. This was achieved through western blot analysis, enabling accurate comparison by adjusting for variations in protein abundance. To validate the methodology applied, mutants with known enzyme activity were used as controls. Positive controls were p.P31L (NC-CAH) [68, 69], p.I173N (SV-CAH) [69, 70], and p.V282L (NC-CAH), and negative control was p.Q319Ter (SW-CAH) [69, 70].

### Western blot

CYP21A2 enzymatic activity values were normalized using CYP21A2/beta actin ratio determined by western blot. Cell lysates were prepared, as previously described [60], and total protein concentration was measured using the PIERCE BSA Protein Assay Kit. A total of 7 µg of protein per sample was separated via sodium dodecyl sulfate-polyacrylamide gel electrophoresis (GenScript) and transferred onto a polyvinylidene fluoride (PVDF) membrane according to standard procedures [60]. For the detection of CYP21A2 protein, a mouse monoclonal anti-FLAG tag antibody (GenScript Cat# A00187, RRID:AB_1720813) was applied at a 1:1000 dilution. β-Actin was used as the internal loading control and detected using a mouse monoclonal anti-β-actin antibody (GenScript Cat. # A00702, RRID:AB_914102) at a dilution of 1:1500. An IRDye 800CW-conjugated donkey anti-mouse secondary antibody (LICORbio Cat# 926-32212, RRID:AB_621847) was used at a 1:10 000 dilution.

### Statistical analysis

All statistical analyses were performed using GraphPad Prism software (v.8.0). To compare the enzymatic activities of multiple mutant variants against the single WT control, a 1-way ANOVA was performed, followed by Dunnett multiple comparisons test. To simultaneously assess the effects of 2 independent variables (variant and substrate) on enzymatic activity, a 2-way ANOVA was used, followed by Šídák multiple comparisons test to compare variant activities for each substrate. A *P*-value of <.05 was considered statistically significant. All experiments were performed in triplicate, with duplicate data points for each replicate and data are presented as mean ± standard deviation.

## Results

### Population genetics and allele frequency analysis

**Figure 2:**
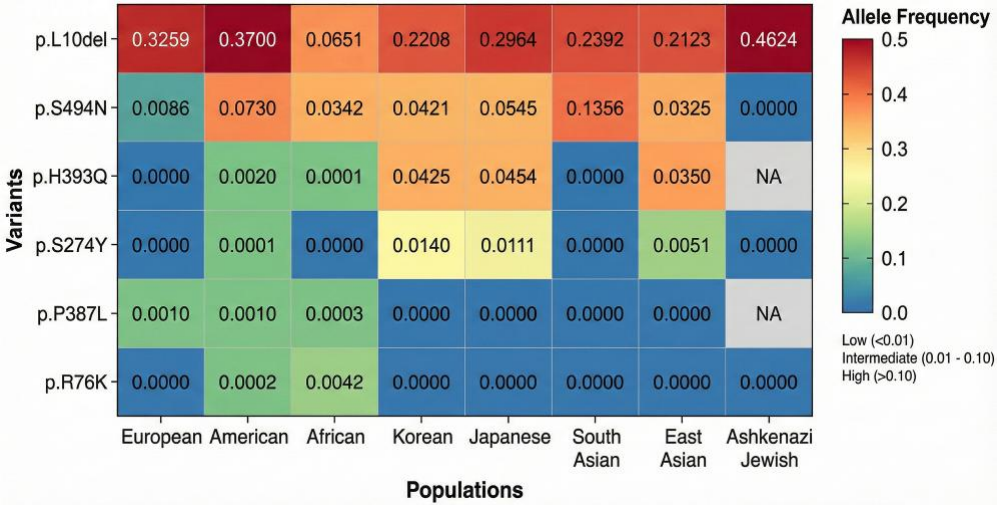
Global allele frequency heat map of *CYP21A2* variants. A heat map visualization of allele frequencies derived from large-scale population databases (gnomAD, ExAC, TOPMed, ALFA, and 1000 Genomes). The color intensity correlates with allele frequency. The p.L10del row displays “hot” colors (orange/red) across multiple populations, indicating frequencies of 20-46%, confirming its status as a common benign polymorphism. p.S494N shows “warm” colors specifically in East Asian and South Asian columns, reflecting an ethnicity-specific polymorphism. The rows for R76K, p.P387L, and p.H393Q are dominated by “cool” colors (blue/green), representing ultra-rare frequencies (<10^−4^) or absence in general populations, a distribution pattern consistent with alleles under negative selection pressure due to pathogenicity. Data are in Table S1 [59].

The first tier of evidence in establishing the pathogenicity of a genetic variant is its frequency within the general population. The prevalence of an allele can serve as a potent filter for pathogenicity; variants causing severe, early-onset recessive disorders like classic CAH are subject to strong negative selection and are therefore expected to be extremely rare. Conversely, alleles present at frequencies significantly higher than the disease carrier frequency are likely benign polymorphisms or associated with very mild, NC phenotypes. We analyzed the allele frequencies of the selected *CYP21A2* variants across multiple large-scale genomic databases, including gnomAD, ExAC, TOPMed, ALFA, and the 1000 Genomes Project (1000G; Fig. 2 and Table S1) [59].

### High-frequency variants indicating benign polymorphisms

Analysis of the variant p.L10del (c.17_19delTGC) revealed frequencies that are fundamentally inconsistent with a pathogenic role in a rare Mendelian disorder. In the 38KJPN (Japanese) database, the allele frequency was observed at 0.29641, and in the ALFA database, it was 0.22776. Furthermore, specific subpopulations showed even higher prevalences, such as 0.338 in Northern Sweden and 0.4624 in Ashkenazi Jewish populations (Fig. 2). The presence of this variant in nearly a third to half of the alleles in certain populations suggests it is a common polymorphism (rs61338903) likely devoid of significant deleterious effects on protein function. Similarly, the p.S494N (c.1481G>A) variant was identified at high frequencies in East Asian populations, with an allele frequency of 0.0545 in Japanese (38KJPN) and 0.0421 in Korean cohorts. The minor allele frequency in the global ALFA dataset was 0.0068, which, while lower than p.L10del, is still well above the expected frequency for a severe pathogenic allele.

The variant p.R76K (c.227G>A) also exhibited frequencies suggestive of a benign nature. In the ALFA database, it was reported at a remarkably high frequency of 5.1 × 10^−4^, although other databases like gnomAD exomes reported a more modest 1.138 × 10^−4^. The high frequency in ALFA (rs368330593) likely reflects specific technical artifacts or population structures in certain datasets. Thus, the African/African American population constitutes the predominant carrier group for the R76K variant. However, the relative contribution of this population differs markedly between datasets, representing 15.1% of all samples in ALFA compared with only 2.5% in gnomAD exomes. This difference in population representation may partly explain the discrepancy in allele frequency estimates between the 2 databases. The overall presence in 1000G at 1.6 × 10^−3^ supports its initial classification as a common variant rather than a disease-causing mutation.

### Rare variants aligning with pathogenic potential

In sharp contrast to the variants described above, the remaining 8 variants, p.E162G, p.S274Y, p.L308V, p.S373N, p.P387L, p.H393Q, p.R401G, and p.R436C, were characterized by extreme rarity or complete absence in general population databases, a hallmark of alleles under purifying selection. The p.L308V (c.922T>G) variant was detected at an ultra-low frequency of 5 × 10^−8^ in gnomAD exomes and 1.9 × 10^−5^ in TOPMed. This scarcity is consistent with a potentially deleterious allele that is efficiently removed from the gene pool or maintained only in heterozygosity. Similarly, p.P387L (c.1160C>T) was found at 2 × 10^−4^ in ExAC and 3 × 10^−4^ in 1000G, frequencies that align with carrier rates for rare recessive traits. The p.R436C (c.1306C>T) variant appeared at 5.3 × 10^−4^ in ALFA and 1.11 × 10^−3^ in ExAC, again falling within the range of potential pathogenicity. Of particular interest is p.H393Q (c.1179C>G). While rare in European-dominated datasets like ExAC (3.12 × 10^−3^), it showed a notable enrichment in Asian populations, with a frequency of 0.0455 in the 38KJPN database and 0.043 in the Korean database. This discrepancy suggests a population-specific allele, potentially a founder mutation associated with a milder, NC phenotype that has escaped strong negative selection in these groups.

A “variant frequency heat map” (Fig. 2) was created to visually summarize the findings from the survey of genomic sequence databases. The heat map displayed a striking dichotomy; the columns for p.L10del are dominated by “hot” colors (red/orange) across almost all populations, visually confirming its status as a ubiquitous polymorphism. The p.S494N row shows “warm” colors specifically in the East Asian and South Asian columns, highlighting its ethnic specificity. Conversely, the rows for p.R76K, p.S274Y, p.P387L, and p.H393Q are predominantly “cool” (blue/green) across European, American, and African populations, indicating near-zero frequencies. This population stratification provided the foundational evidence that p.L10del and p.S494N are likely benign, while the rare variants warranted deeper functional scrutiny to determine their clinical significance.

### Evolutionary conservation analysis (ConSurf)

**Figure 3:**
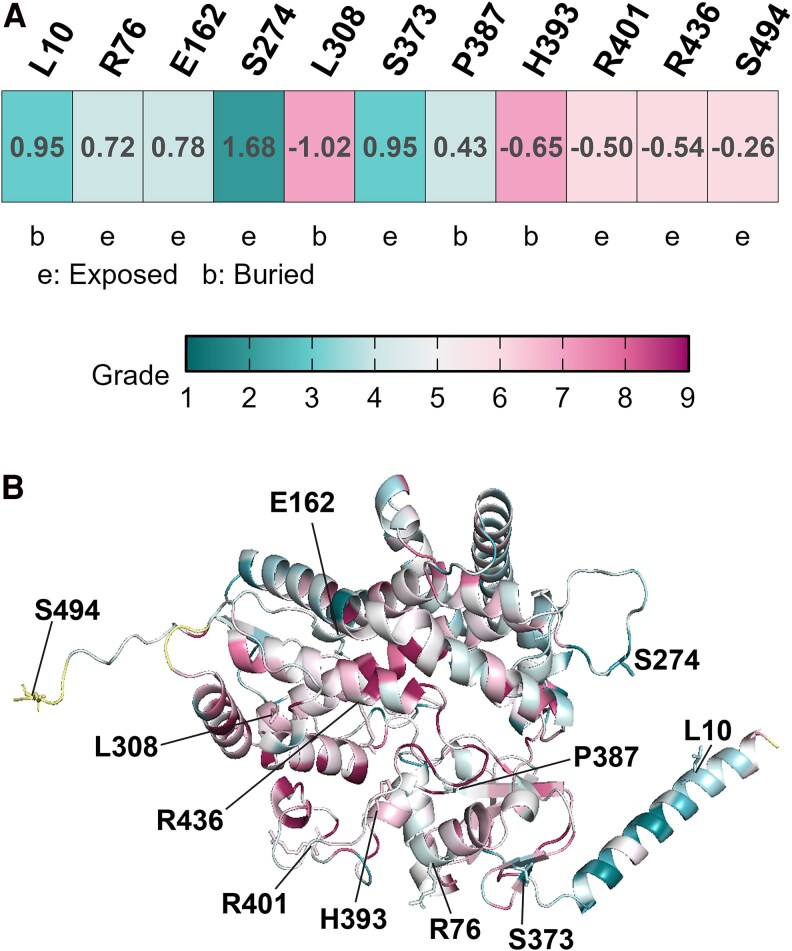
Evolutionary conservation mapping on the CYP21A2 protein structure. (A) ConSurf analysis showing conservation scores for variant residues (Scales 1-9). Highly conserved residues are critical for function/stability. (B) Three-dimensional structural mapping of conservation scores onto the CYP21A2 protein structure. The protein backbone is colored by conservation grade: magenta indicates highly conserved core regions, while cyan indicates variable surface loops. Pathogenic variants p.L308V and p.P387L map to the highly conserved magenta core, suggesting intolerance to mutation. In contrast, benign variants p.L10del and p.S494N map to variable cyan regions on the protein periphery. The heme cofactor is shown as sticks. Data are in Table S2 [59].

To understand the functional constraints acting on the *CYP21A2* gene, we performed an evolutionary conservation analysis using ConSurf (Fig. 3 and Table S2) [59]. This method assigns a conservation score to each amino acid position based on the phylogenetic alignment of homologous sequences from diverse species. Residues that are critical for protein folding, stability, or catalysis are typically highly conserved (score 9), while those in flexible loops or nonfunctional regions tolerate variability (score 1).

The analysis of the 11 variant residues revealed a clear segregation based on their conservation status, as detailed in Table S2 [59]. The highly conserved residues included Leu308, located in the I-helix of the cytochrome P450 structure, received a conservation score of 7. The confidence interval for this score was tight (8,7), indicating high certainty. In the multiple sequence alignment (MSA), Leu308 was present in 100/100 sequences, with only a rare valine substitution observed in 3% of sequences. This strict conservation underscores the structural indispensability of Leu308, which resides in the core of the enzyme and likely plays a role in defining the architecture of the active site. Similarly, Pro387 in the K-L loop scored a 4, with 100/100 sequences retaining this residue. Prolines are unique in their ability to restrict backbone flexibility, often acting as “structural staples.” The absolute conservation of Pro387 suggests that the rigidity it imparts to the K-L loop is critical for the proper folding of the heme-binding pocket. Located on the surface of the L-helix, Arg436 scored a 6. While surface residues are often variable, Arg436 is part of the binding interface for P450 oxidoreductase (POR). Its positive charge is essential for the electrostatic docking with the electron donor protein. The ConSurf analysis confirms that this interaction interface is evolutionarily constrained. His393, a buried residue scored a 7, with high conservation (100/100 sequences in MSA). Its buried nature suggests a role in packing or stabilizing the core through hydrogen bonding or van der Waals interactions.

Among the variable residues (score 1-5), situated in the N-terminal membrane anchor, Leu10 showed high variability (score 3). The MSA data revealed a variety of residues at this position, consistent with the structural role of this region as a simple tether to the endoplasmic reticulum membrane rather than a precise catalytic element. This low conservation supports the population data suggesting p.L10del is benign. The Ser494, located in the disordered C-terminal tail, Ser494 scored a 6. The presence of this residue in only 2/100 sequences in the alignment highlights the lack of evolutionary pressure on the extreme C-terminus. The Arg76 (score 4) located on surface, also showed variability, aligning with the high allele frequency of the p.R76K variant.

### Structural parameters: solvent accessibility and physicochemical properties

To further delineate the structural environment of the variants, we calculated the solvent accessible surface area (SASA) and relative solvent accessibility (RSA) for both the WT and mutant residues. These metrics help distinguish between “buried” residues, which contribute to protein core stability, and “exposed” residues, which interact with the solvent or partner proteins (Table S3) [59]. The physiochemical property analysis reveals distinct structural classes for the variants. Among the buried residues (critical for stability), the His393 residue is almost completely buried, with a total SASA of only 0.80 Å^2^ and an RSA of 0.35%. The side chain contributes 100% of this minuscule accessible area. The mutation to glutamine (Q) results in a negligible change in surface area (ΔRSA = 0.02) but introduces a polar residue into a hydrophobic environment. The buried nature explains why this site is so sensitive to mutation; there is no room for structural accommodation. The Pro387 is also deeply buried (total SASA 4.30 Å^2^, RSA 2.71%). The mutation to leucine slightly decreases the SASA (to 3.90 Å^2^). Being buried in the β-sheet region near the heme, the replacement of the compact proline with the bulkier leucine likely introduces steric strain in the core. Leu308 has a total SASA of 5.08 Å^2^ and an RSA of 2.53%, confirming it is a buried residue. The substitution of valine increases the SASA slightly to 7.28 Å^2^ (ΔRSA = 1.47). As a core residue in the I-helix, its burial is essential for maintaining the hydrophobic integrity of the active site roof.

Among the surface-exposed residues (interaction sites), the R436 is highly exposed, with a total SASA of 87.79 Å^2^ and an RSA of 32.04%. The mutation to cysteine significantly reduces the surface area (total SASA drops to 37.01 Å^2^), leading to a massive negative change in RSA (ΔRSA = −9.88). This drastic reduction in surface presence, combined with the loss of the arginine's charge, directly impairs the protein's ability to interact with the solvent and its redox partner, POR. The R76 residue is also highly exposed (total SASA 189.19 Å^2^, RSA 69.05%). The mutation to lysine maintains the basic charge and results in a minor change in RSA (ΔRSA = −1.66). The high exposure and conservation of physicochemical properties (Arg to Lys) explain why this variant is benign. The E162 is moderately exposed (total SASA 42.81 Å^2^, RSA 19.20%) and its mutation to glycine reduces the SASA to 30.74 Å^2^. The structural parameter analysis reinforces the distinction between core-destabilizing mutations (H393Q, P387L, and L308V) and surface-altering mutations (R436C). The extremely low SASA values for the former group indicate that any mutation at these sites must disrupt the dense protein packing, inevitably leading to stability issues.

### Thermodynamic stability predictions using FoldX and DUET

**Figure 4:**
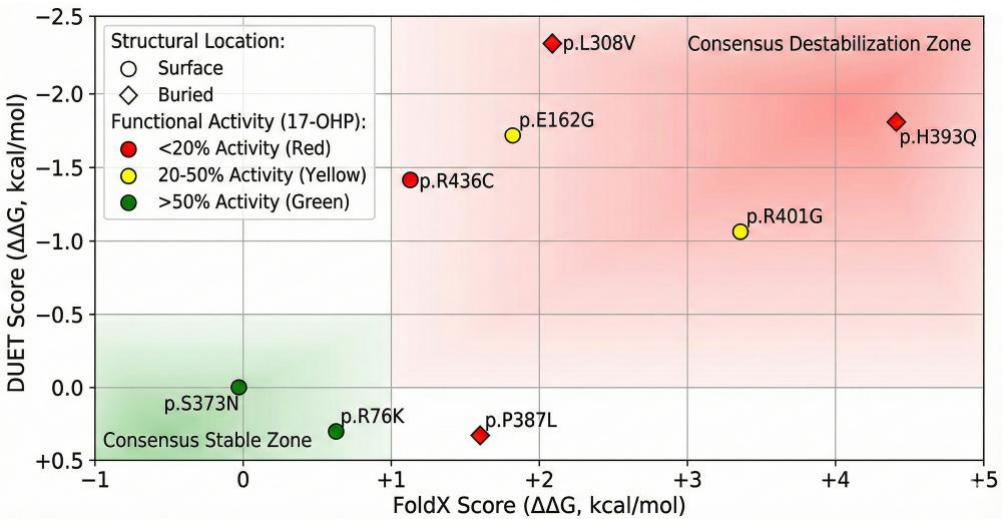
Predicted thermodynamic stability changes (ΔΔ*G*). A comparative bar graph of Gibbs free energy changes (ΔΔ*G*) predicted by FoldX and DUET algorithms. **(***X*-axis) FoldX: Positive ΔΔ*G* values indicate destabilization. p.H393Q and p.R401G show severe destabilization scores (>3 kcal/mol), predicting a likely disruption of the protein fold. **(***Y*-axis) DUET: Negative ΔΔ*G* values indicate destabilization. Consistent with FoldX, p.L308V and p.E162G are predicted to be destabilizing. The benign variants p.S373N and p.R76K exhibit near-neutral scores across both tools, indicating structural tolerability. Data for structural parameters are in Tables S3 and S4 [59].

We utilized computational algorithms, FoldX and DUET, to predict the change in Gibbs free energy of folding (ΔΔ*G*) upon mutation (Fig. 4). These tools provide a quantitative estimate of how much a mutation destabilizes the protein structure compared with the WT (Table S4: FoldX and DUET ΔΔ*G* predictions) [59]. The stability prediction data provided a potential thermodynamic stratification of the variants. Among the severely destabilizing mutations (ΔΔ*G* > 3 kcal/mol), p.H393Q variant exhibited the highest destabilization score in FoldX, with a ΔΔ*G* of 4.47 ± 0.02 kcal/mol. In the DUET analysis, it showed a score of −1.69 kcal/mol (where negative indicates destabilization). Such a high positive FoldX score predicts a loss of stability, likely preventing the protein from folding correctly or targeting it for rapid degradation. This aligns perfectly with its “buried” status; the introduction of glutamine breaks the hydrophobic core. The p.R401G variant also showed severe destabilization, with a FoldX score of 3.42 ± 0.02 kcal/mol (Fig. 4 and Table S4) [59]. Despite being a surface residue, Arg401 likely participates in critical salt bridges that stabilize the tertiary fold. The loss of this interaction energy significantly compromises the protein structure.

Among the moderately destabilizing mutations (ΔΔ*G* < 3 kcal/mol), the p.L308V was predicted to have a destabilization of 2.10 ± 0.07 kcal/mol for FoldX, while DUET predicted −2.15 kcal/mol. This consistent prediction of instability suggests that the L308V mutation weakens the I-helix packing, likely creating a cavity or clash that raises the energy of the folded state. For p.P387L, FoldX yielded a score of 1.50 ± 0.86 kcal/mol, and DUET gave 0.16 kcal/mol. While the FoldX score suggests destabilization, the large standard deviation indicates conformational heterogeneity. The loss of the proline's rigidity likely increases the entropy of the unfolded state, shifting the equilibrium away from the native fold. Interestingly, the p.E162G variant showed a FoldX score of 1.80 ± 0.37 kcal/mol but a DUET score of −1.78 kcal/mol. The conflict suggests complex local dynamics, perhaps involving the flexibility of the D-helix. Among the neutral/stabilizing mutations (ΔΔ*G* ∼ 0), the p.S373N had a FoldX-predicted score of −0.08 ± 0.08 kcal/mol, and for p.R76K, FoldX-predicted score was 0.63 ± 0.10 kcal/mol, indicating that these substitutions are energetically neutral, consistent with their high allele frequencies and likely benign nature. However, in silico prediction tools cannot make an accurate estimation of multiple biological factors that drive protein expression and are only an estimate of potential stability calculation with currently available tools. A combination of prediction tools along with molecular dynamics (MD) simulations is employed and needs to be compared with observed protein expression levels.

### Molecular dynamics simulations for assessing dynamic stability

When a mutation is introduced in an enzyme, it may have several effects depending on the nature of the mutated residue. Effects comprise steric effects caused by an increased size of the side chain of the mutated residue, electrostatic effects caused by changed protonation, local or even long-range conformational effects due to changed preferences of conformations of side chain and backbone (Gly and Pro) residues. Thus, the structure of a mutated CYP21A2 enzyme may be different or behave differently compared with the WT CYP21A2 enzyme and, accordingly, may present a different binding site to a substrate molecule, which may lead to altered biological activity.

**Figure 5:**
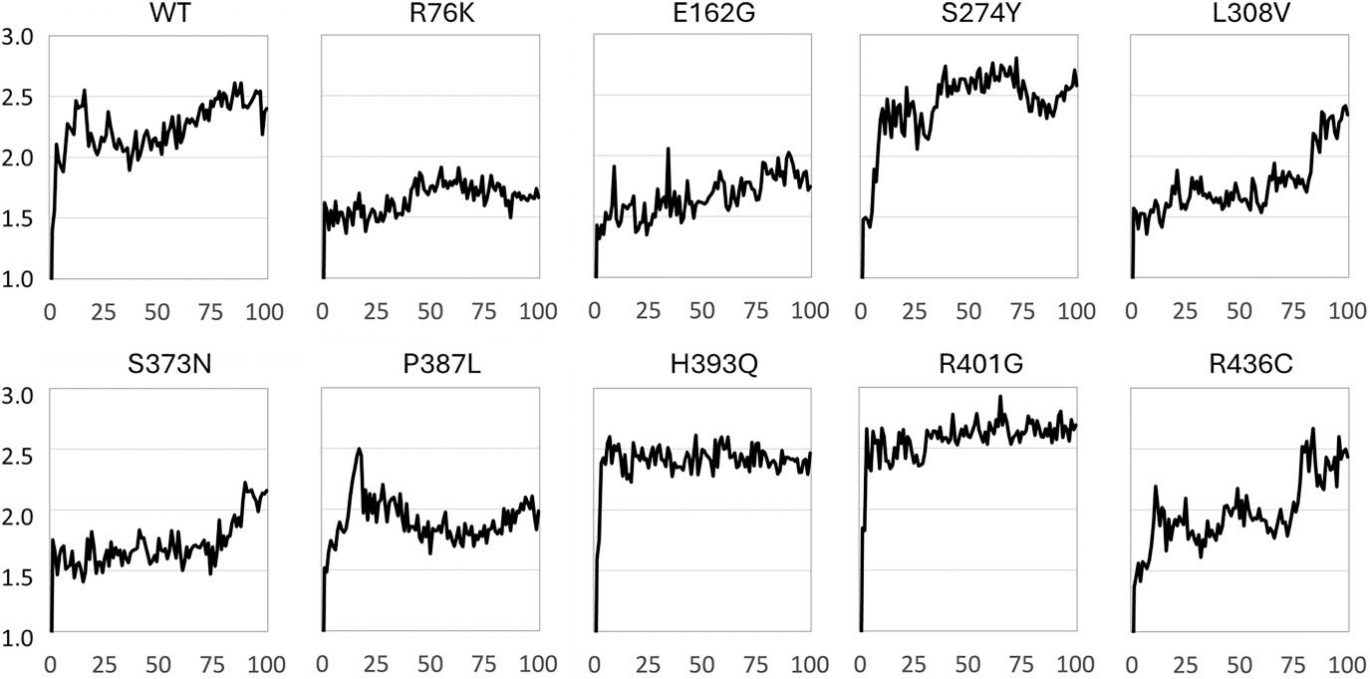
Dynamic structural stability of CYP21A2 protein variants assessed by Cα RMSD from molecular dynamics simulations. Cα RMSD (Å) is plotted as a function of simulation time (in ns) for WT CYP21A2 and 9 missense variants. Trajectories for 100 ns MD simulations of enzyme–substrate (progesterone) complexes are shown.

To identify whether the variants are able to bind a substrate and form stable enzyme–substrate complexes, we performed MD simulations (100 ns) on the WT CYP21A2 protein complex with progesterone as well as on the progesterone complexes with the 9 mutated enzymes (Fig. 5). The RMSD for the WT complex fluctuated in the range 2.0 to 2.5 Å suggesting that the WT complex slightly flexible. The S274Y had a similar profile, whereas the H393Q and R401G variants immediately changed to structures with RMSD = 2.4 and 2.6 Å, respectively, and remained stable for the remaining simulation time. Two complexes, R76K and E162G, were significantly more stable than the WT complex. The remaining variants displayed different degrees of drifting, and we are cautious to conclude too much from these trajectories, under the conditions employed in this study. While longer and multiple MD simulations would be required to make more firm conclusions based only on computational analysis, our study employed both computational and biological experiments to derive conclusions about each variant.

### In silico pathogenicity prediction: consensus analysis

**Figure 6:**
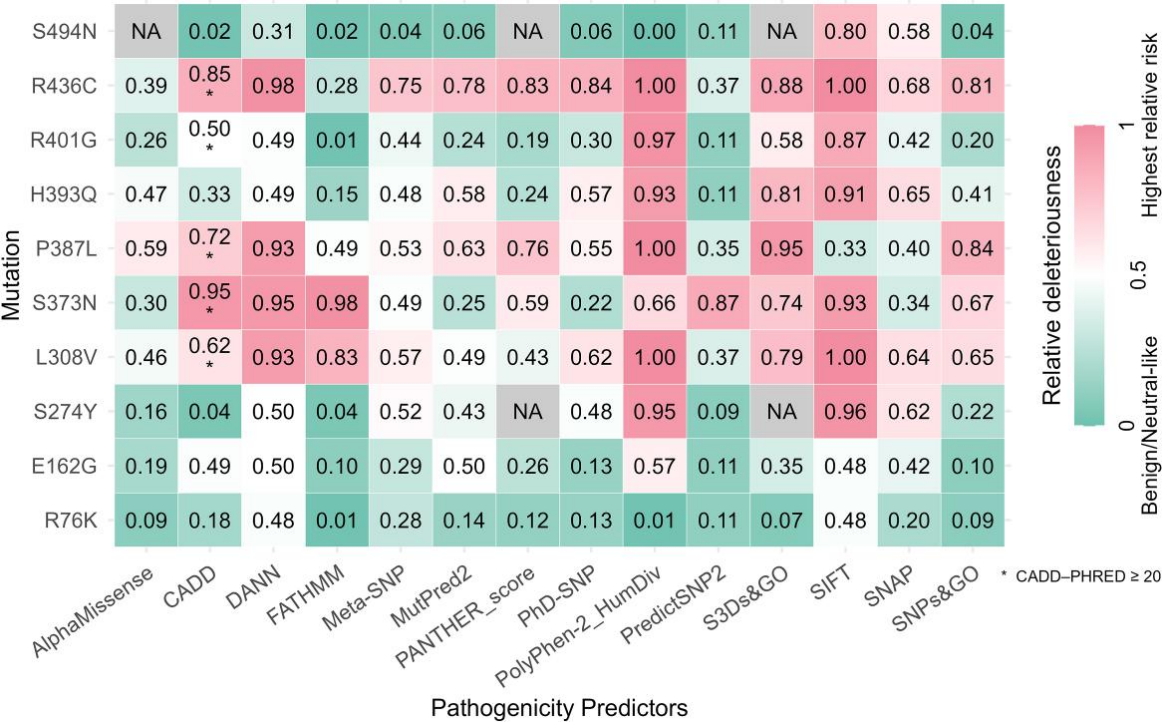
In silico pathogenicity prediction consensus. A heat map aggregating scores from 14 computational prediction tools (including CADD, PolyPhen-2, and SIFT; color version of the figure is in supplementary material). (Red) High pathogenicity: The columns for p.L308V, p.P387L, and p.R436C are universally red, indicating a consensus “deleterious” prediction. (Green) Benign: The columns for p.S494N and p.R76K are predominantly green/blue, indicating a “benign” consensus. (Mixed) VUS: p.E162G shows conflicting scores (green and red), consistent with its mild functional impairment and association with NC CAH. Exact numbers are given in Table S5 [59]. Data were normalized to fit outputs of different tools in one image to a range of 0 to 1.

We utilized a battery of 14 computational prediction tools to generate a consensus view of variant pathogenicity (Fig. 6). The results, summarized in the “In silico pathogenicity prediction scores” (Table S5 and Fig. 6), provide a preliminary basis for functional classification [59]. Among the unanimous pathogenic consensus, p.P387L was classified as “deleterious,” “damaging,” or “disease” by almost every tool. PolyPhen-2 gave it a score of 1.00 (probably damaging), CADD score was 24.7, p.H393Q was also universally considered disease causing with a PolyPhen-2 score 0.93, CADD 16.45, and DANN 0.98. The p.R436C also consistently scored as pathogenic (CADD 26, PolyPhen-2 1.00, and SIFT 0). The p.L308V had a high pathogenic score across the board (CADD 22.4, PolyPhen-2 1.0).

For the unanimous benign consensus, the p.S494N was classified as “benign” or “neutral” by almost all tools (CADD 0.16, PolyPhen-2 0.003) and p.R76K was also consistently benign (CADD 12.56, PolyPhen-2 0.01). Mixed results (VUS) were obtained for p.E162G, which generated conflicting predictions. It was called “possibly damaging” by PolyPhen-2 (0.57) but “tolerated” by SIFT (0.52). The CADD score was 19.73. This pattern is typical of variants with mild or partial loss of function, often associated with NC CAH.

### Functional characterization: protein expression and enzymatic activity

The evidence for pathogenicity comes from the direct measurement of protein function in vitro. We expressed the variants in HEK293T cells and measured both their steady-state protein levels (western blot) and their enzymatic activity using radiolabeled progesterone and 17-OHP.

### Protein expression levels (western blot)

The western blot data (Fig. S2) revealed significant differences in protein stability [59]. Reduced stability was observed for p.L308V and p.R436C which showed markedly reduced protein levels, at ∼46% and 49% of WT, respectively. This reduction confirms that these mutations destabilize the protein, likely leading to increased turnover or aggregation. For p.L308V, this matches the FoldX and MD data perfectly. Surprisingly, p.H393Q (159%) and p.P387L (122%) showed protein levels higher than WT. Despite the FoldX prediction of severe destabilization, these proteins accumulate in the cell. This discrepancy further highlights the need for experimental validation rather than relying only on computational predictions.

### Enzymatic activity analysis

**Figure 7:**
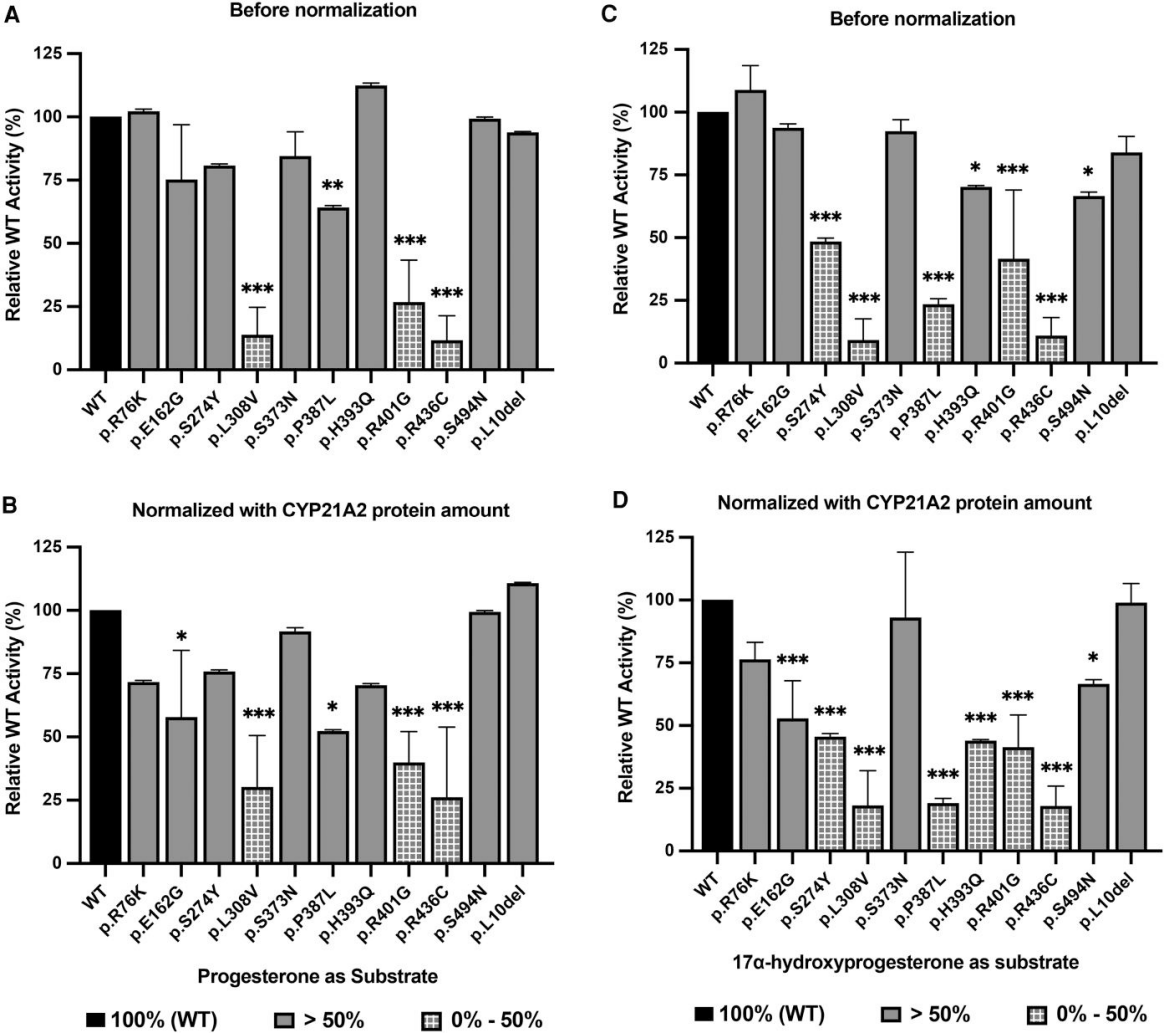
Enzymatic activity: progesterone and 17-OHP conversion. Bar graphs depicting the 21-hydroxylation of progesterone. (A) Raw activity (% of WT). (B) Activity normalized to protein expression. The p.L308V and p.R436C variants show severe loss of function (<30%), while p.H393Q and p.R401G display moderate impairment (40-70%), characteristic of NC alleles. (C and D) Bar graphs depicting the conversion of 17-OHP, a critical step for cortisol synthesis. (Note: StAR-mediated cholesterol transfer to pregnenolone is the actual rate-limiting step.) (C) Raw activity. (D) Normalized activity. The variants p.L308V, p.P387L, and p.R436C exhibit severe catalytic failure (<20% activity), potentially classifying them as pathogenic alleles associated with simple virilizing CAH. p.L10del retains ∼99% activity, confirming its benign status. The dissociation between progesterone and 17-OHP activity for p.P387L highlights substrate-specific impairment. Data are in Table S6 [59].

Enzymatic activity was measured for the conversion of progesterone (to 11-DOC) and 17-OHP (to 11-deoxycortisol). The results are presented as a percentage of WT activity, normalized to protein expression. To ensure accurate interpretation of phenotypic severity, these activities were benchmarked against known control variants assayed under identical conditions: p.P31L and p.V282L (associated with NC-CAH, typically yielding 20-50% activity), p.I173N (associated with SV-CAH, typically yielding 1-5% activity), and p.Q319Ter (associated with SW-CAH, yielding ∼0% activity) (Fig. 7, Table S6) [59]. Furthermore, all calculated percentage conversions have been rounded to whole numbers to accurately reflect the precision limits of the in vitro assay.

#### Severe loss of function (pathogenic—simple virilizing)

**Figure 8:**
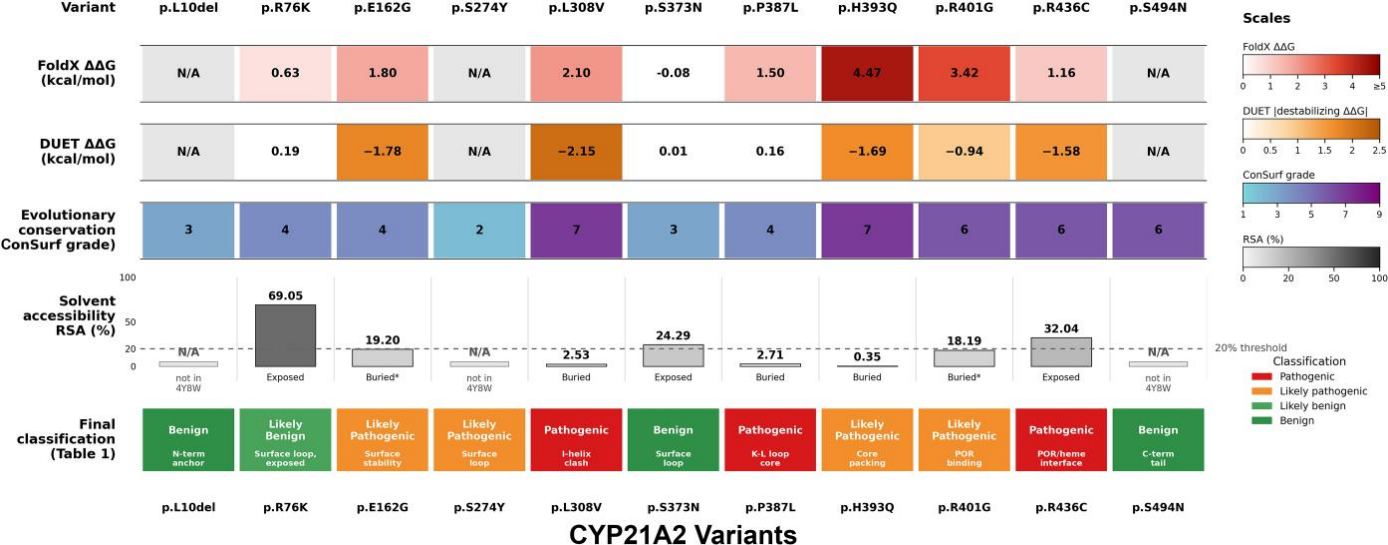
Summary of stability, functional, and enzyme assays.

The p.L308V, an Ultra-rare (5 × 10^−8^ in gnomAD) is a destabilizing ΔΔG 2.1 kcal/mol) and MD simulations suggest drifting RMSD (Fig. 5). It had 17-OHP Activity of 18% ± 11% (*P* < .001) while activity with progesterone as a substrate was 30% ± 17% (*P* < .001) of WT. The p.L308V retains <20% of normal activity for the key cortisol precursor. This places it firmly in the pathogenic category, likely causing SV CAH (Fig. 8). This resolves the conflict with the null p.L308fs mutation; p.L308V is distinct but still severe. The p.P387L had activity with 17-OHP as substrate only 19% ± 1.3% (*P* < .001) of the WT, while with progesterone it showed an activity of 52% ± 0.4% compared with WT. Interestingly, protein levels are high (122%), suggesting the formation of inactive aggregates that escape immediate degradation but cannot function.

This difference in activities with 2 different common substrates of CYP21A2 enzyme shows a significant substrate dissociation. While progesterone activity is preserved at 50% (NC range), 17-OHP activity is reduced to ∼19% (SV range). Since cortisol deficiency drives CAH, the low 17-OHP activity dictates the clinical phenotype, classifying this as a pathogenic variant likely causing SV or severe NC CAH. The p.R436C had a 17-OHP activity of 18% ± 6.5% (*P* < .001) and with progesterone it showed an activity of 26% ± 23% (*P* < .001) compared with WT. With <20% activity, p.R436C is a severe allele (Fig. 8). The impairment of both substrates confirms that the disruption of the POR binding site (predicted by structure) reduces the electron transfer efficiency required for catalysis.

#### Moderate loss of function (likely pathogenic: NC)

**Figure 9:**
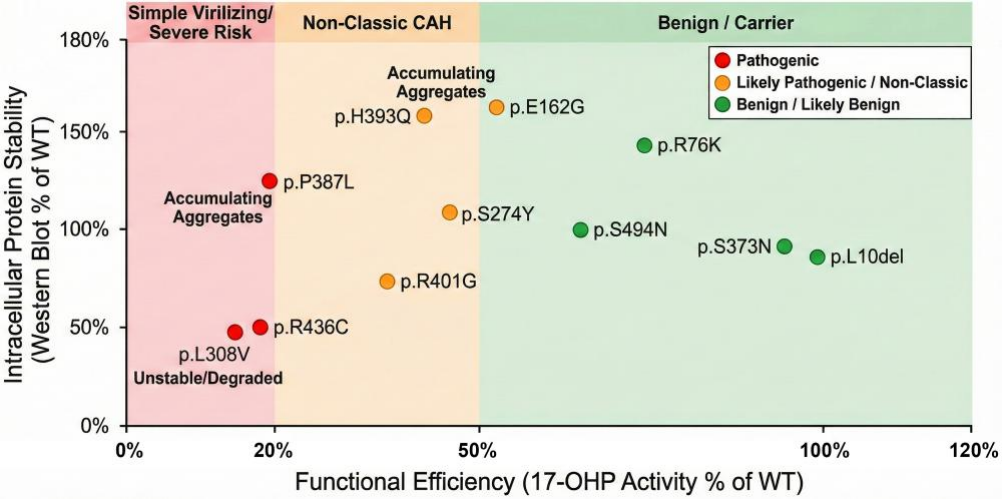
Categorization of CYP21A2 protein variants.

The p.H393Q showed a 17-OHP activity: 44% ± 0.3% (*P* < .001) and with progesterone its activity was 70% ± 0.4% of WT. The activity is reduced to the 40% to 50% range. This is the classic hallmark of an NC allele (Fig. 9). It is not a null mutation, but it is insufficient for normal stress responses and protein levels remained high (159%). For p.R401G a 17-OHP activity of 41% ± 10% and with progesterone an activity of 40% ± 10% was observed. This consistent ∼40% activity across substrates confirms p.R401G as a pathogenic mutation associated with the NC phenotype. For p.E162G we saw a 17-OHP activity of 53% ± 12%, indicating a mild NC allele or a very rare polymorphism with functional impact (Fig. 9).

#### Benign function

This trinucleotide deletion resulting in missing Leu 10 residue in the N-terminal membrane anchor is ubiquitous. Allele frequencies reach 46% in Ashkenazi Jewish populations and 30% in Japanese cohorts. Structurally, Leu10 resides in the flexible transmembrane tether, a region with low conservation scores (ConSurf score 3) that does not participate in catalysis. Functional assays confirm this, showing ∼99% of WT activity for 17-OHP and ∼110% for progesterone. p.S373N located on a surface loop, has a neutral free energy change ΔΔ*G* −0.08 kcal/mol). Functional activity at ∼93% is indistinguishable from the WT. The conservative substitution of arginine to lysine in p.R76K maintains the positive charge on the protein surface. It is highly exposed (RSA ∼ 69%) and energetically neutral (ΔΔ*G* ∼ 0.6 kcal/mol). Functional assays show ∼76% activity. While ALFA reports a high frequency (0.51), gnomAD reports it as rare (10^−4^), suggesting database artifacts or specific population clusters. The biochemical data supports a benign status. The p.S494N variant shows distinct ethnic stratification, being common (>5%) in East Asian populations but rare in others. It is located in the disordered C-terminal tail (residue 494), which has poor electron density in crystal structures and low conservation (ConSurf score ∼4). Functional activity is ∼67% for 17-OHP, a reduction that is statistically significant but clinically irrelevant given the high population frequency in healthy individuals. It functions as a benign polymorphism with slightly reduced efficiency.

### Structural mechanisms of dysfunction

**Figure 10:**
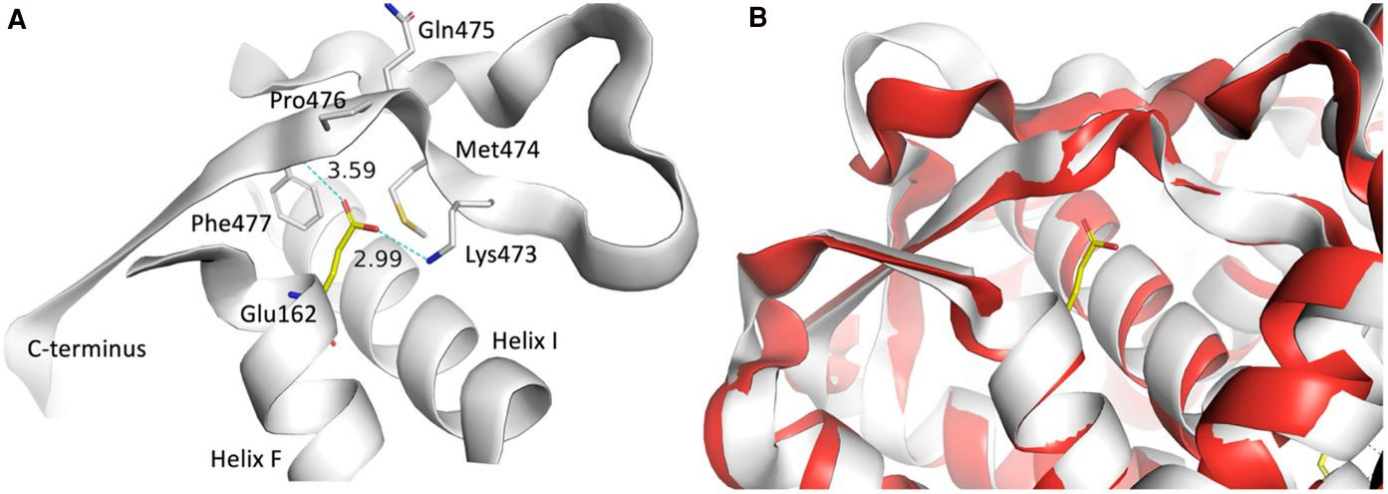
Structural consequence of the p.E162G substitution in CYP21A2 protein. (A) WT CYP21A2 protein with Glu162 and environment formed by Helix F, Helix I, and the C-terminus. Hydrogen bonds from Lys473 and Phe477 are shown as dotted lines. (B) Initial (white) and final (red) frames from MD simulation of CYP21A2 E162G shown as a cartoon through the Cα atoms. The Glu162 from the WT CYP21A2 protein structure has been added to the figure to illustrate the point of mutation.

To explain why these mutations cause enzymatic failure, we analyzed the specific molecular interactions in the modeled mutant structures. The mutation of glutamate to glycine in the p.E162G variant represents a major structural modification with at least 2 likely consequences, destabilization of the 3D structure due to lack of hydrogen bonds to the negatively charged side chain in Glu162 and increased flexibility associated with a glycine residue compared with any alpha-substituted residue (Fig. 10). The Glu162 residue is located at the beginning of Helix F with its side chain pointing across Helix I and forming hydrogen bonds with the side chain nitrogen atom in Lys473 and the backbone nitrogen atom in Phe477 located in the C-terminal part of the CYP21A2 protein structure (Fig. 10A). An MD simulation of the E162G variant showed no significant conformational change of the C-terminal part (Fig. 10B).

**Figure 11:**
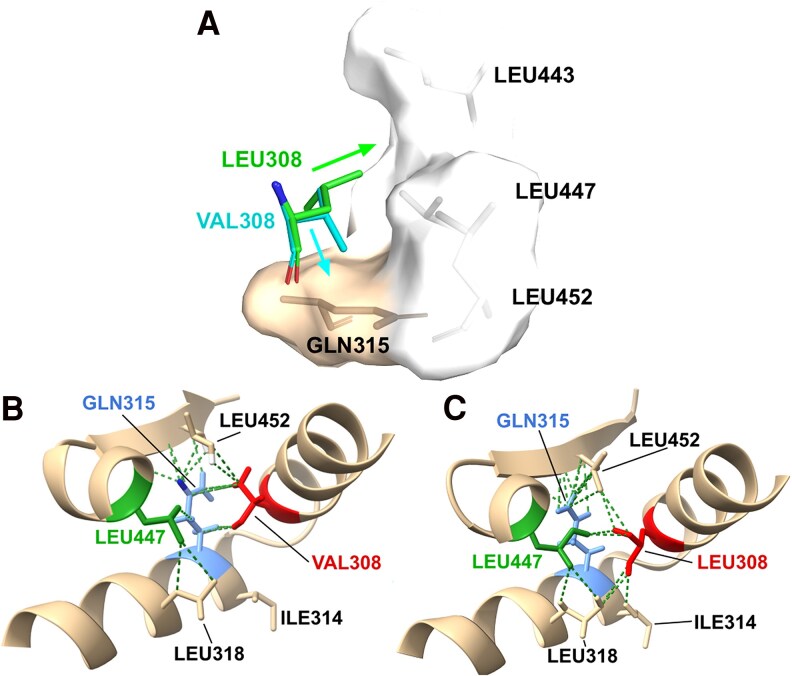
Structural mechanism of p.L308V: I-Helix destabilization. (A) The WT Leu308 residue forms a tight hydrophobic packing network within the conserved I-helix. (B) The p.L308V mutation introduces a valine. Modeling shows that the specific rotamer required for valine creates a steric clash with Gln315 and a packing void (cavity) due to the shorter side chain. (C) This disruption distorts the I-helix geometry, which is essential for proton delivery to the heme iron, thereby uncoupling the catalytic cycle.

In the WT structure, Leu308 is a keystone residue in the I-helix (Fig. 11). Its bulky isobutyl side chain interlocks with Leu318, Ile314, Leu447, and Leu452, creating a hydrophobic network that stabilizes the “kink” in the I-helix essential for oxygen activation (Fig. 11A). The p.L308V mutation introduces a valine, which while chemically similar, has a different shape (β-branched). Our modeling reveals that the specific rotamer of valine required to fit in the helix backbone induces a steric clash with Gln315 (Fig. 11B). Simultaneously, the shorter side chain creates a hydrophobic cavity in the packing network with L447/L452. The combination of a steric clash (distorting the backbone) and a packing cavity (destabilizing the core) disrupts the precise geometry of the I-helix. Since the I-helix is responsible for delivering protons to the heme iron, this disruption uncouples the catalytic cycle, explaining the 18% residual activity.

**Figure 12:**
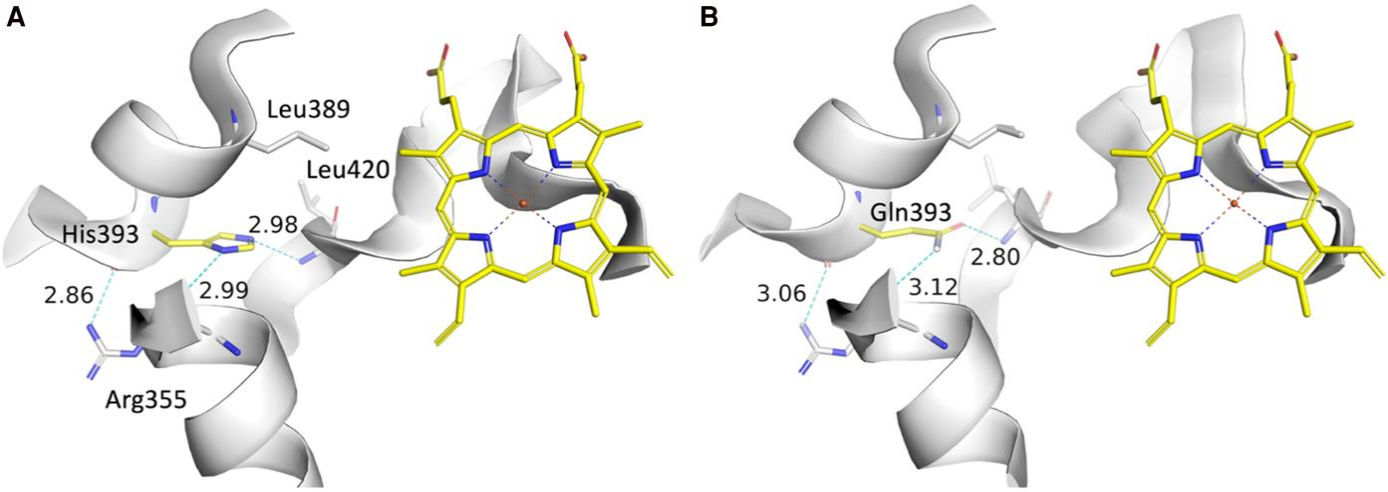
Comparison of CYP21A2 WT protein and H393Q interactions. (A) The imidazole ring in the WT structure makes hydrogen bonds with the Arg355 side chain and the Leu420 backbone nitrogen. (B) In the Gln393 variant, the amide side chain maintains an identical hydrogen network with the carbonyl oxygen and nitrogen atoms being hydrogen-bond acceptor and donor, respectively. The (B) structure is the final structure after a 100 ns MD simulation.

In the p.H393Q variant, a histidine is replaced by a glutamine, which must be characterized as a conservative mutation since the side chains are equal in length, and both contain a hydrogen-bond acceptor and a hydrogen-bond donor (Fig. 12). In the WT CYP21A2, protein His393 imidazole ring donates a hydrogen bond to the backbone oxygen atom in Arg355 and accepts a hydrogen bond from the backbone nitrogen atom in Leu420 (Fig. 12A). The hydrogen bonding pattern is maintained in the mutated form and remains stable during an MD simulation (Fig. 12B).

**Figure 13:**
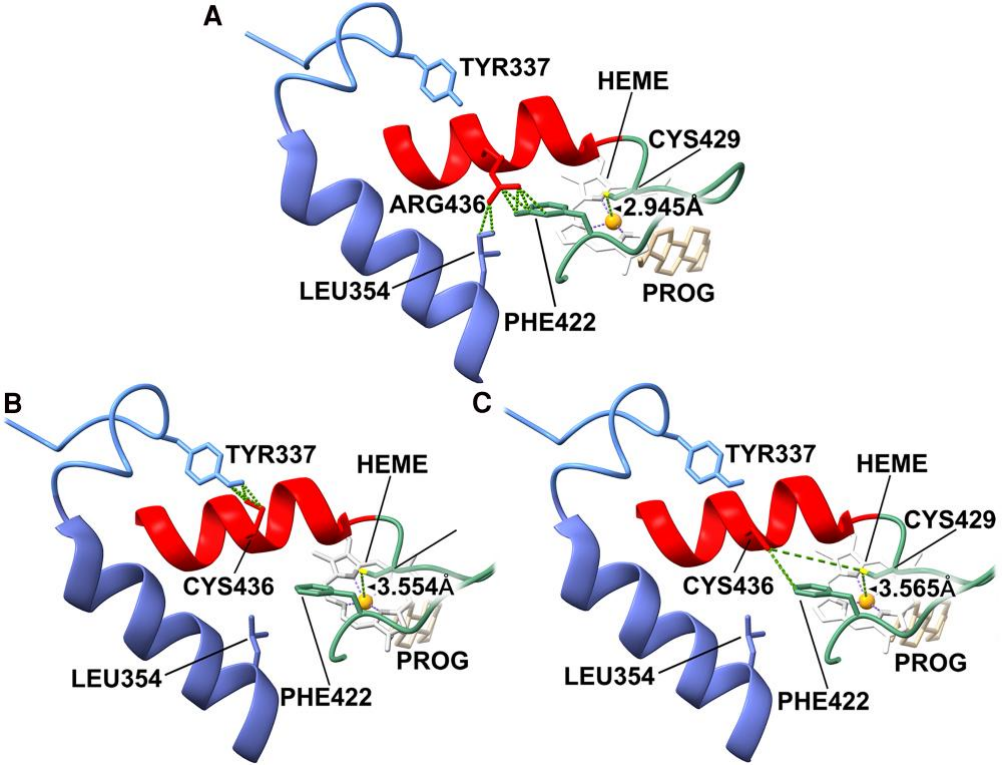
Structural mechanism of p.R436C: heme-ligand interface disruption. (A) In WT CYP21A2 protein, Arg436 forms a surface hydrogen-bond network with Leu354 and Phe422, stabilizing the loop containing the heme-ligating Cysteine 429. (B) The p.R436C mutation abolishes this network. (C) The loss of the Arg436 “strut” increases the flexibility of the Cys429 loop, causing the Cys429 sulfur to shift away from the heme iron (distance increases to ∼3.6 Å). This alteration in the ligand field impairs electron transfer from P450 oxidoreductase, causing severe activity loss despite the surface location of the mutation.

Arg436 acts as a molecular strut on the protein surface (Fig. 13). In WT, the long Arg436 side chain forms a crucial hydrogen-bond network with Leu354 (J-helix) and Phe422 (Fig. 13A). This network stabilizes the loop containing Cys429, the thiolate ligand that holds the heme iron in place. The mutation to cysteine (p.R436C) removes this long reach (Fig. 13B). The short cysteine cannot bridge the gap to L354 or F422. Without the Arg436 residue, the Cys429-loop becomes flexible. Our model predicts that the Cys429 sulfur atom shifts away from the heme iron, increasing the bond distance from 2.9 Å (optimal) to 3.6 Å (dysfunctional; Fig. 13C). This increased distance weakens the ligand field strength, altering the redox potential of the heme and making electron transfer from POR inefficient. This explains why a surface mutation causes such a profound (18% activity) catalytic defect.

## Discussion

**Figure 14:**
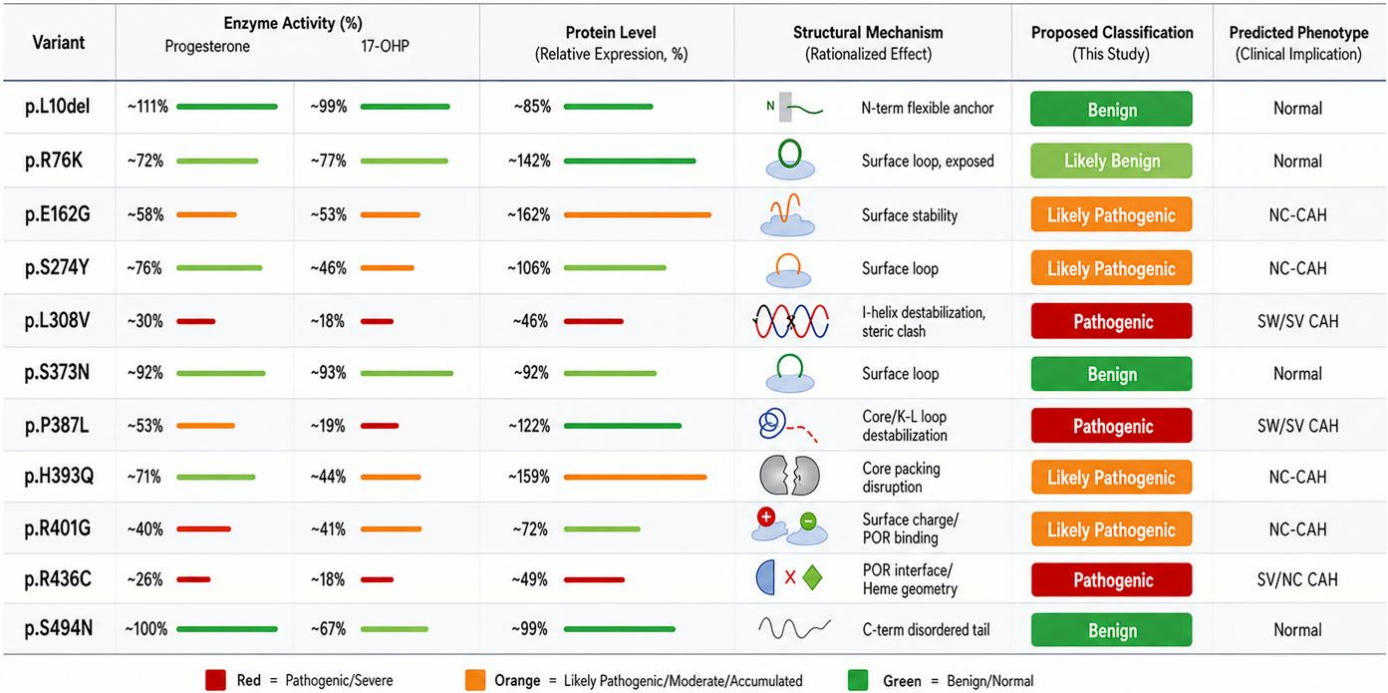
Revised classification scheme. A proposed “Traffic Light” diagnostic algorithm. Red (pathogenic/SV): p.L308V, p.P387L, p.R436C. (suggestion: carrier screening, high CAH risk). Orange (likely pathogenic/NC): p.H393Q, p.R401G, and p.E162G (suggestion: monitor for late-onset symptoms). Green (benign): p.L10del, p.S494N, p.R76K, and p.S373N (suggestion: exclude from panels). Activity values are percentages relative to WT CYP21A2 enzyme activity. Protein level reflects relative expression in heterologous systems.

This study represents a functional and structural interrogation of 11 *CYP21A2* gene variants, potentially resolving their status from “uncertain” to clinically actionable categories. By synthesizing data from global population frequencies, evolutionary biology, thermodynamic stability, and direct enzymatic assays, we have constructed a comprehensive profile for each variant (Fig. 14). The primary clinical imperative of this work was to clarify the pathogenicity of variants with conflicting or absent data. The clinical interpretation of variants at codon 308 has historically been fraught with ambiguity due to the presence of the p.L308fs frameshift allele. This frameshift, resulting from a microdeletion, leads to a truncated, nonfunctional protein and is a well-documented cause of classic SW CAH. However, the missense variant p.L308V (c.922T>G) has frequently been conflated with the null allele in clinical databases, leading to conflicting reports regarding its severity. Our study provides a functional and structural separation of these 2 entities. While p.L308fs is a null mutation, our data demonstrates that p.L308V is a stable but severely catalytically compromised variant. Structural modeling reveals that the substitution of the leucine isobutyl side chain with the valine isopropyl group, a seemingly conservative change, has significant geometric consequences. The valine side chain induces a specific steric clash with Gln315 and creates a hydrophobic cavity within the highly conserved I-helix. This helix is not merely a structural scaffold; it contains the conserved acid-alcohol pair essential for proton delivery to the heme-bound oxygen intermediate. The distortion of this helix uncouples the proton transfer pathway, explaining the ∼18% residual activity observed for 17-OHP. This finding firmly places p.L308V in the pathogenic category, likely associated with the SV phenotype rather than the SW form seen with the frameshift. Clinically, this distinction is vital: patients carrying p.L308V may have sufficient residual aldosterone production to avoid neonatal SW crises but remain at high risk for postnatal virilization, necessitating a distinct monitoring protocol.

A central tenet of protein engineering suggests that mutations in the hydrophobic core are destabilizing, while surface mutations are generally tolerated unless they disrupt specific binding sites. Our characterization of p.R436C challenges this simplification in the context of CYP21A2 protein (Fig. 14). Despite its high solvent accessibility (RSA > 30%) and significant distance from the active site, p.R436C exhibits a catastrophic loss of enzymatic function (<20% activity), comparable to core-destabilizing mutations like p.L308V. We elucidate a novel “remote control” mechanism for this dysfunction. Arg436 acts as a molecular anchor on the protein surface, maintaining a crucial hydrogen-bond network with Leu354 and Phe422. This network rigidifies the internal loop containing Cys429, the proximal thiolate ligand that coordinates the heme iron. Our structural modeling predicts that the p.R436C mutation severs this network, increasing the entropy of the Cys429 loop and causing the sulfur atom to shift away from the iron (Fe–S distance increases from 2.9 to ∼3.6 Å). This alteration in coordination geometry likely perturbs the redox potential of the heme, impairing the transfer of electrons from POR. Thus, p.R436C represents a “functional” mutation where the protein fold is intact (as evidenced by stable MD trajectories and western blot), but the catalytic engine is uncoupled from its fuel source. This finding underscores the critical importance of analyzing surface VUS for their role in the obligate protein-protein interaction interfaces that drive P450 function. The p.P387L showed core instability, with a drifting RMSD profile in 100 ns MD simulation (structural unraveling) and low 17-OHP activity (19%) and is reclassified as pathogenic (SV/NC). The kinetic instability suggests that patients might have a “temperature-sensitive” phenotype, where fever or stress precipitates a drop in enzyme function, triggering adrenal crises.

The p.H393Q and p.R401G show intermediate activity (40-45%) and significant destabilization scores and are reclassified as likely pathogenic (NC). These variants are likely responsible for late-onset CAH. The stark divergence in allele frequencies observed for p.H393Q and p.S494N emphasizes the necessity of ancestry-matched reference populations in genetic diagnosis. While p.H393Q is effectively absent in European cohorts (ExAC/gnomAD), our analysis reveals a significant enrichment in East Asian databases (Japanese and Korean frequency ∼4.5%). Our functional assays characterize p.H393Q as an NC allele with ∼44% residual activity. The combination of high frequency and moderate functional impairment strongly suggests a “founder effect” in East Asian populations, where the allele has drifted to higher prevalence, possibly maintained by the lack of strong negative selection against the milder NC phenotype. Similarly, p.S494N appears as a common polymorphism in Asian cohorts (>5%) but is rare elsewhere. The identification of these population-specific alleles has immediate clinical implications. For a patient of East Asian descent presenting with signs of late-onset CAH (eg, hirsutism and fertility issues), p.H393Q should be a primary target for genotyping. Conversely, its detection in a European patient would be an anomaly requiring careful segregation analysis. Furthermore, the high frequency of the benign p.S494N variant in Asian populations mandates its exclusion from standard diagnostic panels to prevent false-positive diagnoses and unnecessary anxiety for carriers. These findings argue for the implementation of ethnicity-specific filtering in CAH diagnostic pipelines.

Equally important is the removal of benign variants from disease panels to prevent false positives. The p.L10del and p.S494N are widespread polymorphisms with near-WT activity (∼100% and ∼67%, respectively) and variable conservation. They should be classified as benign and removed from diagnostic consideration for CAH. The p.R76K and p.S373N are also benign, showing high exposure, low conservation, and normal activity. The distinct mechanisms identified, core destabilization (L308V, P387L) vs surface uncoupling (R436C), can potentially be explored for therapeutic possibilities (Fig. 14).

While this study utilized a robust HEK293T system, limitations exist. The ratio of POR to P450 in kidney cells differs from the adrenal cortex, which could affect the absolute *V*_max_ values. Furthermore, we focused on the classic pathway; future studies should investigate the impact of these variants on the 11-oxygenated androgen pathway (eg, 11-ketotestosterone), which is increasingly recognized as a key driver of androgen excess in CAH. Overall, this study elevates the status of p.L308V, p.R436C, p.P387L, p.H393Q, and p.R401G to pathogenic/likely pathogenic, while potentially clearing p.L10del and p.S494N.

A limitation of this study is the reliance on heterologous expression systems and the absence of direct clinical data from patients harboring these specific variants. As noted in prior literature, in vitro activities can exhibit variability, and absolute activity percentages must be interpreted cautiously alongside established phenotypic controls. Furthermore, our population analysis suggests that certain pathogenic VUS may be enriched in specific ethnic groups (eg, p.H393Q in East Asian cohorts). While the fundamental molecular consequence of a mutation remains constant regardless of ethnicity, the clinical manifestation (CAH vs NCAH) in a patient ultimately depends on the combination of alleles (compound heterozygosity) they inherit. Therefore, while a variant cannot be “SV-causing” in one ethnicity and “NC-causing” in another inherently, the prevalent phenotype in a region will depend on the local allele pool. The structural and functional data provided here serve as a foundational tool to guide future clinical correlations when patients carrying these rare variants are identified in the clinic.

## Conclusion

This study addresses a critical gap in the molecular diagnosis of 21-OHD by providing a functional and structural characterization of 11 *CYP21A2* gene variants previously classified as VUS or having conflicting interpretations. By integrating population data, evolutionary conservation, thermodynamic stability predictions, and direct enzymatic assays, we have successfully reclassified these variants into clinically actionable categories ranging from benign to pathogenic (Fig. 14 and Table 1). Our results exonerate the variants p.L10del, p.S494N, p.R76K, and p.S373N. The high population frequency of p.L10del and p.S494N, combined with their retention of near-WT enzymatic activity (66-99%), confirms they are benign polymorphisms that should be excluded from diagnostic panels to prevent false-positive diagnoses.

**Table 1:** Summary of evidence for *CYP21A2* gene variants

Variant	Initial ClinVar	Activity (Prog), %	Activity (17OHP), %	Protein level, %	Structural mechanism	Proposed classification	Predicted phenotype
p.L10del	Benign	∼111	∼99	∼85	N-term flexible anchor	Benign	Normal
p.R76K	VUS	∼72	∼77	∼142	Surface loop, exposed	Likely benign	Normal
p.E162G	VUS	∼58	∼53	∼162	Surface stability	Likely pathogenic	NC-CAH
p.S274Y	N/A	∼76	∼46	∼106	Surface loop	Likely pathogenic	NC-CAH
p.L308V	Likely pathogenic	∼30	∼18	∼46	I-helix destabilization, steric clash	Pathogenic	SW/SV CAH
p.S373N	Likely pathogenic	∼92	∼93	∼92	Surface loop	Benign	Normal
p.P387L	Likely pathogenic	∼53	∼19	∼122	Core/K-L loop destabilization	Pathogenic	SW/SV CAH
p.H393Q	Likely benign	∼71	∼44	∼159	Core packing disruption	Likely pathogenic	NC-CAH
p.R401G	VUS	∼40	∼41	∼72	Surface charge/POR binding	Likely pathogenic	NC-CAH
p.R436C	Likely benign	∼26	∼18	∼49	POR interface/heme geometry	Pathogenic	SV/NC CAH
p.S494N	Benign	∼100	∼67	∼99	C-term disordered tail	Benign	Normal

Conversely, we provide robust evidence for the pathogenicity of p.L308V, p.P387L, and p.R436C. The p.L308V variant, often confused with the p.L308fs frameshift, is confirmed here as a distinct, severe missense mutation. Structural modeling demonstrates that it destabilizes the I-helix via steric clashes and cavity formation, resulting in a residual activity of ∼18% for 17-OHP, consistent with the SV phenotype. Similarly, p.P387L causes severe core destabilization and dynamic instability, while p.R436C disrupts the critical interface with P450 oxidoreductase, impairing electron transfer despite being located on the protein surface. Both retain <20% activity and are classified as pathogenic.

Furthermore, we identified p.H393Q, p.R401G, and p.E162G as alleles associated with moderate loss of function (40-55% residual activity). These variants are likely drivers of the NC CAH phenotype, with p.H393Q showing a specific enrichment in Asian populations, suggesting a founder effect. In summary, this work refines the genotype–phenotype correlation map for CAH. These findings will immediately improve the accuracy of genetic counseling and clinical management for patients carrying these specific *CYP21A2* alleles.

## Data Availability

All data are available in the manuscript or in the supplementary materials at https://doi.org/10.7910/DVN/BA7FZZ [59].

## Abbreviations

21-OHD: 21-hydroxylase deficiency
17-OHP: 17-hydroxyprogesterone
CAH: congenital adrenal hyperplasia
NC: nonclassic
POR: P450 oxidoreductase
RMSD: root mean square deviation
RSA: relative solvent accessibility
SASA: solvent accessible surface area
SW: salt-wasting
VUS: variants of uncertain significance
WT: wild type
